# Modulating the default mode network with deep TMS: a proof-of-concept framework with potential relevance to circuits implicated in lack of awareness of cognitive decline

**DOI:** 10.3389/fnagi.2026.1742759

**Published:** 2026-04-22

**Authors:** Youssef Bellaali, Dominika Sulcova, Adriana Salatino, Laurence Dricot, Bernard Jimmy Hanseeuw, John L. Woodard, Adrian Ivanoiu

**Affiliations:** 1 Institute of Neuroscience, Université Catholique de Louvain, Brussels, Belgium; 2 Wayne State University, Detroit, MI, United States

**Keywords:** Alzheimer disease, anosognosia, continuous theta burst stimulation (cTBS), default mode network (DMN), fMRI, posterior cingulate cortex (PCC), TMS

## Abstract

**Introduction:**

Self-referential processes are suspected to rely on the default mode network (DMN), particularly the posterior cingulate cortex (PCC). The right PCC, has been implicated in self-awareness, with neuroimaging studies showing its involvement in anosognosia in Alzheimer’s disease. However, causal evidence linking right PCC activity to DMN synchrony in humans remains scarce. This study examined whether transient modulation of the right PCC using continuous theta-burst deep transcranial magnetic stimulation (deep TMS) alters DMN connectivity in healthy adults.

**Aim:**

To assess whether transient inhibition of the right PCC using continuous theta-burst deep transcranial magnetic stimulation (deep TMS) modulates DMN functional connectivity in healthy adults.

**Methods:**

Twelve right-handed healthy participants (aged 26–30 years) underwent two randomized sessions: deep TMS targeting the right PCC and a sham condition. Resting-state fMRI was acquired 10–20 min post-stimulation. Connectivity changes were assessed through (1) a correlation-matrix analysis between the right PCC and 78 regions of the Brainnetome atlas, and (2) a whole-brain seed-based paired *t*-test. For the whole-brain analysis, statistical significance was determined using cluster-level Monte-Carlo correction (*p* < 0.05).

**Results:**

The region-based correlation matrix showed reduced connectivity between the PCC and three DMN-related regions (right precuneus, right inferior parietal gyrus, left superior temporal gyrus), although these did not survive multiple-comparison correction. The whole-brain analysis revealed eight clusters with significantly decreased connectivity after stimulation compared to sham, including six key DMN hubs (bilateral precuneus, bilateral supramarginal gyri, right angular gyrus, left posterior superior temporal gyrus).

**Conclusion:**

Deep TMS of the right PCC induced a transient desynchronization of DMN connectivity, supporting a causal contribution of the right PCC to large-scale self-referential network integrity. While no behavioral evaluation was performed at this stage, these results provide a proof-of-principle that transient modulation of the right PCC can alter DMN synchrony, opening the way for future studies combining deep TMS with metacognitive and self-awareness assessments to directly investigate awareness mechanisms in healthy aging and neurodegenerative disease.

## Introduction

### The posterior cingulate cortex and the default mode network: a neural substrate of self-referential processing

The default mode network (DMN) is a large-scale brain network involved in internally directed cognition, including autobiographical memory, internal mentation, and self-referential thought (Lagercrantz and Changeux, 2009; Hellyer et al., 2014). Among its core hubs, the posterior cingulate cortex (PCC) plays a pivotal role in self-focused attention and the maintenance of self-related information during rest and introspection (Lou et al., 2004; Kircher et al., 2000; Kjaer et al., 2002). Neurodevelopmental data support the involvement of DMN nodes, including the PCC, in the emergence of narrative self-representation (Lagercrantz and Changeux, 2009). During rest or self-reflection, the PCC consistently shows increased activation, highlighting its role in internal awareness and cognitive self-processing (Hellyer et al., 2014; Lou et al., 2004).

Converging evidence suggests that the PCC contributes to self-awareness processes and introspective evaluation. PET and fMRI studies have demonstrated PCC engagement during self-referential judgments and reflective self-awareness (Lou et al., 2004; Kircher et al., 2000; Kjaer et al., 2002). Its central anatomical and functional position in the DMN enables it to integrate self-relevant information with autobiographical memory systems (Cavanna and Trimble, 2006). Consequently, disruption of PCC function is hypothesized to impair insight into one’s own cognitive abilities and internal states.

### Disturbances of self-awareness in AD: anosognosia and lack of awareness of cognitive decline

Disturbances of self-awareness are a well-documented feature of Alzheimer’s disease (AD). Clinically defined anosognosia, most frequently observed at moderate to severe stages of the disease, refers to a marked and persistent lack of awareness of one’s own cognitive and functional impairments (Spalletta et al., 2012). By contrast, lack of awareness of cognitive decline represents a broader and more graded construct, emphasizing awareness of cognitive change and allowing quantification along a continuum, for example through patient–informant discrepancy measures (Starkstein, 2014).

Importantly, converging clinical and experimental evidence suggests that anosognosia in AD reflects, at least in part, a breakdown in the ability to accurately self-evaluate cognitive performance and functional limitations (Starkstein, 2014). This impairment of self-evaluation is suspected to be in part related to metacognitive monitoring processes, which, in non-clinical contexts, are typically assessed using behavioral indices such as confidence–accuracy relationships (Cosentino et al., 2007). From this perspective, anosognosia can be conceptualized as a clinically manifest and severe disruption of self-evaluative mechanisms that, in milder or earlier forms, could be captured by experimental measures of metacognition.

### PCC–DMN dysfunction and impaired awareness in AD

Neuroimaging studies consistently demonstrate abnormal DMN connectivity in AD compared with healthy aging (Greicius, 2008; Greicius et al., 2004), with early involvement of core DMN hubs, including the posterior cingulate cortex (PCC), demonstrated across imaging modalities (Minoshima et al., 1997; Vannini et al., 2017b; Perrotin et al., 2015; Johnson et al., 1998). Functional connectivity and metabolic studies further suggest that PCC dysfunction is associated with impaired awareness in both prodromal and established AD (Vannini et al., 2017a,b; Perrotin et al., 2015; Antoine et al., 2019; Bastin et al., 2021; Guerrier et al., 2018). Notably, after the hippocampus, the PCC is among the earliest regions affected by AD pathology, exhibiting hypometabolism (Minoshima et al., 1997; Johnson et al., 1998), amyloid deposition (Buckner et al., 2005), and structural degeneration (Vannini et al., 2017b; Karas et al., 2007). Reduced PCC metabolism predicts progression to dementia (Bailly et al., 2015) and correlates with the severity of anosognosia (Perrotin et al., 2015; Vannini et al., 2017a; Therriault et al., 2018). Together, these clinical and neurobiological findings underscore the early vulnerability of PCC-centered DMN circuitry and motivate causal perturbation approaches aimed at transiently modulating PCC–DMN coupling *in vivo*.

Several studies further suggest a preferential involvement of the right PCC in awareness-related processes. Neuroimaging experiments report stronger right-hemispheric PCC engagement during reflective self-awareness tasks (Kjaer et al., 2002), and the right PCC has been proposed to play a leading role in mental self-representation (Lou et al., 2004). Consistent with this view, right PCC hypometabolism has been associated with impaired self-awareness in AD and mild cognitive impairment (Vannini et al., 2017b; Perrotin et al., 2015; Antoine et al., 2019; Therriault et al., 2018), providing a rationale for selectively targeting the right PCC in experimental paradigms.

### From correlational evidence to causal network perturbation

Despite this substantial body of evidence, current knowledge remains largely correlational. In clinical populations, it is difficult to establish a causal link between PCC dysfunction and disturbances of awareness, as neural alterations in AD are multifocal and confounded by widespread pathology, disease progression, and compensatory reorganization (Starkstein, 2014; Mograbi et al., 2009; Morris and Mograbi, 2013; Bellaali et al., 2021; Seeley et al., 2009). Experimental approaches allowing direct and reversible perturbation of PCC activity are therefore required to test mechanistic hypotheses.

Transcranial magnetic stimulation (TMS) enables transient modulation of cortical excitability in humans (Hallett, 2007), and continuous theta-burst stimulation (cTBS) provides a reliable method to induce inhibitory neuromodulatory after-effects (Huang et al., 2005). However, because of its deep anatomical location, the PCC cannot typically be reached by classical TMS coils. Advances in deep TMS technology now allow stimulation of deeper neural structures, providing a unique opportunity to transiently modulate PCC activity in healthy subjects.

Importantly, the present study is explicitly framed at the network level and builds on a conceptual distinction between related but non-equivalent constructs. In Alzheimer’s disease, anosognosia is commonly defined as a clinically significant lack of awareness of one’s own cognitive and functional impairments. Accumulating evidence indicates that this clinical phenomenon reflects, at least in part, a breakdown in the ability to accurately self-evaluate cognitive performance and everyday functional limitations, a process closely related to metacognitive monitoring mechanisms. In this sense, anosognosia in AD can be conceptualized as a severe and clinically manifest disruption of self-evaluative processes that, in milder or earlier forms, could be captured by experimental measures of metacognition (e.g., discrepancies between objective performance and subjective appraisal).

At a more fundamental level, self-referential or internally directed cognition refers to a class of mental processes commonly associated with default mode network (DMN) activity and provides a neural context within which self-evaluation and awareness-related processes unfold. While self-referential cognition, metacognition, and anosognosia are theoretically and empirically related, they remain dissociable constructs operating at different layers; from network to clinical level.

### Scope of the present study and conceptual framework

Consistent with this distinction, the present study does not include behavioral or metacognitive measures and therefore does not directly assess metacognition or anosognosia. Rather, it focuses on transient modulation of PCC-centered DMN connectivity as a putative neural substrate supporting self-evaluative processes that are disrupted in anosognosia.

The primary aim of this study was to establish an experimental model of transient DMN disruption by targeting the right posterior cingulate cortex (PCC) using deep TMS in healthy young adults. Demonstrating that inhibitory stimulation of the right PCC alters PCC-centered DMN functional connectivity provides a proof-of-concept for causal perturbation of a network critically implicated in awareness-related disturbances in Alzheimer’s disease. This network-level framework is intended as a foundational step, preceding future work that will directly investigate how PCC-centered DMN modulation impacts time-resolved behavioral indices of metacognitive monitoring.

## Methods

### Participants

Twelve young healthy volunteers (6 females, 6 males; age range 26–30 years, mean age 28) took part in the study. All participants were right-handed, as confirmed using the Edinburgh Handedness Inventory, and had no contraindication to transcranial magnetic stimulation (TMS) or MRI. Exclusion criteria included a personal or family history of epilepsy and the use of chronic medication.

A narrow young-adult age range was selected to ensure greater homogeneity in brain functional connectivity and to minimize age-related confounding factors, such as variability in neurovascular coupling, early microvascular lesions, or preclinical neurodegenerative changes, which are more common in older individuals.

All participants provided written informed consent in accordance with the Declaration of Helsinki, and the study protocol was approved by the Ethical Committee of UCLouvain (Ref: 2019/19SEP/410, B403201941608).

### Design

Participants completed three study visits, each scheduled 1 week apart. The first session consisted of acquiring a high-resolution structural MRI used to generate an individual 3D brain model for neuronavigation. The structural MRI was inspected to exclude the presence of any brain lesion that would contraindicate continued participation in the experiment.

During the two subsequent visits, participants received either real deep TMS targeting the right posterior cingulate cortex (rPCC) or sham stimulation, in a randomized and counterbalanced order. No pre-stimulation resting-state fMRI run was acquired at each visit; therefore, comparisons are based on post-stimulation scans obtained in separate sessions. To mitigate potential session-related confounds, the study used a randomized, counterbalanced within-subject crossover design, and we additionally assessed whether the sham–stimulation differences were influenced by session order (stimulation-first vs. sham-first). Thus, half of the participants received real stimulation during the second visit and sham during the third, whereas the remaining half received sham first followed by real stimulation. In both sessions, stimulation was followed by an fMRI examination.

Resting-state fMRI was acquired 10–20 min later (mean 15.2 ± 3.4 min) after the end of stimulation. During this interval, participants remained seated quietly and were instructed to stay relaxed. MRI acquisition began with a T1-weighted scan, followed by resting-state fMRI. During the resting-state acquisition, participants lay with their eyes closed and were instructed to let their thoughts wander naturally without focusing on anything in particular.

### Deep TMS protocol

Continuous theta-burst stimulation (cTBS) was used as the neuromodulation protocol. This patterned stimulation approach is known to induce inhibitory after-effects on cortical excitability lasting up to approximately 1 h after stimulation (Huang et al., 2005). The cTBS train consisted of bursts of three pulses at 50 Hz, repeated every 200 ms (5 Hz) for 40 s, yielding a total of 600 pulses.

Stimulation intensity was set at 80% of the motor threshold, which was determined individually for each participant. The motor threshold was established by delivering single-pulse TMS over the lower-limb motor cortex, located at a cortical depth comparable to the rPCC, while recording surface electromyography (EMG) responses from the left tibialis anterior muscle. Surface EMG electrodes were positioned over the tibialis anterior muscle belly with an inter-electrode distance of 2 cm, and a ground electrode was placed ipsilaterally over the ankle. Electrode placement followed the European recommendations for surface EMG (Cacchio et al., 2009; Hermens et al., 2000). The motor threshold was defined as the lowest stimulation intensity producing motor evoked potentials (MEPs) exceeding 50 μV in ≥50% of 10 consecutive trials (Rossini et al., 1994).

Deep TMS was delivered using a double-cone coil (MagVenture DB80), designed to engage relatively deep midline cortical regions. The right posterior cingulate cortex (rPCC) target was defined from MNI coordinates (4, −53, 42) and projected to each participant’s native anatomical space using their individual T1-weighted MRI. Subject-specific 3D anatomical reconstructions were used for accurate target localization and scalp projection. Coil placement was guided with neuronavigation (Visor 2, ANT Neuro), which provides real-time tracking of coil position/orientation and visualization of the estimated induced electric field to support targeting. Across sessions, coil position and orientation were optimized to maximize the estimated induced field at the intended rPCC target and were continuously monitored to ensure reproducible placement.

To preserve participant blinding, sham stimulation was delivered using the same coil and the same scalp location corresponding to the neuronavigated rPCC target, but with the coil rotated by 90° relative to the scalp surface to minimize effective cortical stimulation while preserving the acoustic click and cutaneous sensations. In the active condition, the coil was positioned tangentially over the individualized rPCC scalp projection, with orientation and placement continuously monitored using neuronavigation (Visor 2, ANT Neuro), at this location, stimulation is far from the motor cortex and does not evoke peripheral muscle responses. Participants were blind to condition; the experimenter administering stimulation was not.

### MRI scanning

All participants underwent three MRI sessions consisting of one high-resolution three-dimensional (3D) T1-weighted structural scan and two resting-state functional MRI (rs-fMRI) runs. Data were acquired on a 3 T MRI system (SIGNA Premier, GE Healthcare, USA) equipped with a 48-channel head coil at the Cliniques universitaires Saint-Luc (UCLouvain, Brussels, Belgium).

#### Structural imaging

A whole-brain 3D T1-weighted MPRAGE sequence (1 mm^3^ isotropic) was acquired with inversion time (TI) = 900 ms, repetition time (TR) = 2188.16 ms, echo time (TE) = 2.96 ms, flip angle (FA) = 8°, field of view (FOV) = 256 × 256 mm^2^, matrix = 256 × 256, 156 slices, slice thickness = 1 mm, no gap; total scan time = 5 min 36 s. Structural images were inspected to exclude brain lesions that would contraindicate continued participation.

#### Functional imaging

Resting-state fMRI data were collected with multiband echo-planar imaging (multiband factor = 3; ARC = 2 parallel imaging), FOV = 220 × 220 mm^2^, matrix = 110 × 110, TE = 30 ms, FA = 90°, 64 ascending, interleaved slices, slice thickness = 2 mm; TR = 1,500 ms. Each run comprised 250 whole-brain volumes (duration = 6 min 15 s).

### MRI pre-processing and data analysis

The MRI data were analyzed using BrainVoyager [Version 2.8, Brain Innovation, Maastricht, The Netherlands]. Preprocessing of the resting-state data consisted of a linear trend removal to exclude scanner-related signal drift, temporal high-pass filter to remove frequencies lower than 0.005 Hz and correction for head movements using a rigid body algorithm for rotating and translating each functional volume in 3D space. Data were also corrected for time differences in the acquisition of the different slices. Data were co-registered with their 3D T1-weighted scans and normalized in the MNI space. All co-registrations were verified and movement corrections were optimized, using a sinc interpolation. Because spontaneous low-frequency fluctuations are not exclusively BOLD-related fluctuations, but are also contaminated by non-neural signals (i.e., artifacts), several additional pre-processing steps were added to remove these undesirable sources of variance. Regression analyses were performed to remove artifacts due to residual motion (the six movement regressors were obtained via rigid body correction of head motion as implemented in BrainVoyager) and changes in ventricles (the signal from a ventricular region of interest defined in each subject) which captures a substantial portion of non-neuronal physiological noise, particularly higher-frequency fluctuations related to cardiac and respiratory activity. The final data were smoothed in the spatial domain (Gaussian filter: Full Width at Half Maximum = 5 mm). We used BrainVoyager and a customized Matlab code (The Mathworks) to calculate cross-correlations between the average time-course signals, extracted from 78 regions of interest (ROIs) intersected with the individual brain mask of each subject. These regions were derived from the Brainnetome atlas (Mograbi et al., 2009). This resulted in 3,081 pairs of functional connectivity per subject. In a second time, we generated first-level connectivity maps for each participant, between the seed region and all voxels in the whole brain, and entered for the second-level analysis, these connectivity maps in a whole-brain paired *t*-test to investigate the effect between the two conditions. Second-level whole-brain inference. Within our seed-based framework, connectivity maps derived from the rPCC seed were interpreted as a functional representation of PCC-centered DMN connectivity, consistent with the established role of the PCC as a core hub of the default mode network. Atlas-based DMN parcels were used as an anatomical comparator for visual interpretation rather than as a strictly equivalent representation of the seed-based maps. Sham vs. rPCC-stimulation seed-based connectivity maps were compared using a voxelwise paired *t*-test. An initial voxel-wise threshold of *p* < 0.05 (uncorrected) was used to define candidate clusters. Correction for multiple comparisons was then performed using a cluster-size thresholding approach implemented in BrainVoyager, based on Monte Carlo simulations estimating the probability of observing clusters of a given size by chance (Kriegeskorte et al., 2006). Using this procedure (1,500 permutations), the minimum cluster-size threshold was set to 187 mm^3^.

## Results

**Figure 1:**
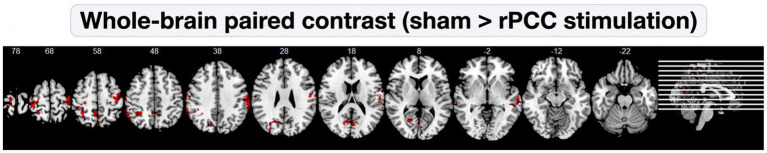
Whole-brain paired contrast of seed-based connectivity (sham > rPCC stimulation). Red clusters indicate regions showing lower functional connectivity with the rPCC seed during active stimulation than during sham. Axial slices are displayed in MNI space across successive superior levels, and the sagittal panel indicates slice positioning. Eight clusters survived whole-brain cluster-extent Monte Carlo correction (cluster-wise *p* < 0.05; minimum cluster size = 187 mm^3^). Six of these clusters were located within canonical DMN territories, namely the right angular gyrus, right supramarginal gyrus, left supramarginal gyrus, right precuneus, left precuneus, and left posterior superior temporal gyrus; two additional significant clusters were located outside the core DMN (see Table 1 for peak coordinates and anatomical labels). All significant effects were in the same direction, reflecting reduced coupling with the rPCC under stimulation relative to sham.

We computed, for each participant and for each condition (real rPCC stimulation vs. sham), the Pearson correlation between the BOLD time course in the right posterior cingulate cortex (rPCC; Brainnetome A31) and the time courses of the 78 default mode network (DMN) subregions defined in the Brainnetome atlas. Condition-wise correlation coefficients were compared with paired *t*-tests (see Figure 1).

**Figure 2:**
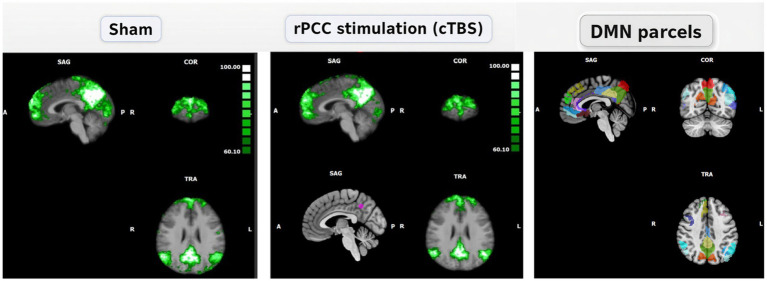
Seed-based connectivity maps under sham **(A)** and right PCC stimulation (cTBS) **(B)**, with anatomical reference to canonical DMN parcels. Three orthogonal views are shown for each condition (sagittal, coronal, axial; orientation labels A/P and R/L). Green overlays represent thresholded group-level functional connectivity maps obtained using the right posterior cingulate cortex (rPCC; Brainnetome A31) as the seed region and are displayed here as a functional representation of PCC-centered DMN connectivity. In panel B, the stimulation target is indicated on the sagittal view by a purple marker corresponding to the neuronavigated rPCC target (MNI: 4, −53, 42). For anatomical comparison, canonical DMN parcels are displayed in an adjacent panel. These atlas-based parcels are provided as an anatomical reference to facilitate visual comparison with the seed-based maps and should not be interpreted as strictly equivalent to the functional connectivity maps. Resting-state fMRI acquisition started 15.2 ± 3.4 min after stimulation; in each session, a T1-weighted scan preceded rs-fMRI acquisition.

Three DMN parcels exhibited significant uncorrected condition differences (sham > stimulation), indicating reduced connectivity following stimulation: Left superior temporal gyrus (A22C; *p* = 0.040; Cohen’s *d*ᶻ = 0.67; *Δ*Fisher-z = 0.069), Right inferior parietal gyrus (A40C; *p* = 0.008; *d*ᶻ = 0.93; Δ = 0.056), and Right precuneus (A7M; *p* = 0.032; *d*ᶻ = 0.71; Δ = 0.057). However, none of these parcel-wise effects survived correction for multiple comparisons across the 78 DMN ROIs, including Bonferroni familywise control and Benjamini–Hochberg FDR (all *q* > 0.05). These ROI-wise findings are therefore reported as exploratory/descriptive and interpreted in relation to the primary whole-brain cluster-corrected results (see Figure 2).

**Figure 3:**
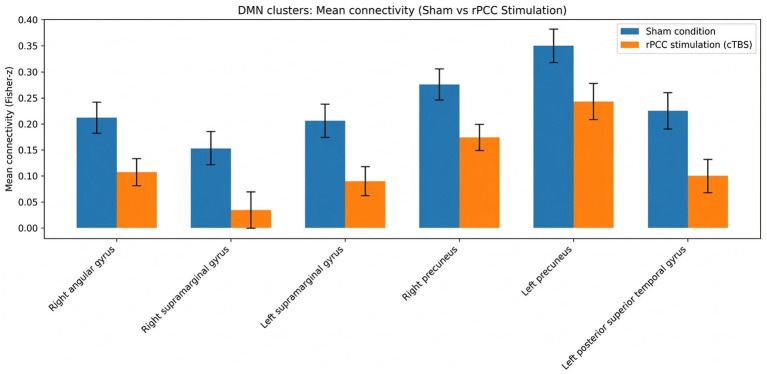
Mean connectivity values within the six significant DMN clusters identified in the whole-brain contrast. Bar plots show mean Fisher-*z* transformed connectivity values between the right posterior cingulate cortex (rPCC) seed and the six significant clusters located within canonical DMN territories: right angular gyrus, right supramarginal gyrus, left supramarginal gyrus, right precuneus, left precuneus, and left posterior superior temporal gyrus. Blue bars indicate the sham condition and orange bars the active rPCC stimulation (cTBS) condition. Error bars represent the standard error of the mean (SEM) across participants (*n* = 12). This figure is intended as a descriptive summary of the direction and magnitude of the group-level effect within the DMN clusters identified by the whole-brain seed-based analysis. Across all six clusters, mean connectivity was lower following stimulation than following sham.

Secondly, a voxelwise paired *t*-test contrasted sham vs. rPCC-stimulation seed-based connectivity maps, using cluster-extent Monte Carlo correction for multiple comparisons (cluster-size threshold = 187 mm^3^). Eight clusters survived correction, all showing lower correlation with the rPCC seed (A31) during stimulation relative to sham. Six of the significant clusters were located within canonical DMN territories—namely the right angular gyrus, right supramarginal gyrus, left supramarginal gyrus, right precuneus, left precuneus, and left posterior superior temporal gyrus—and two additional clusters outside the core DMN (see Table 1 for peak coordinates and anatomical labels). All significant effects were in the same direction (stimulation < sham), and the cluster-level results survived the Monte Carlo multiple-comparison correction. The primary contrast yielded a large within-subject effect (Cohen’s *d*_h_ = 0.933; *t* (11) = 3.233), corresponding to an approximate two-tailed achieved power of ~0.85 at *α* = 0.05, and the 95% CI for the mean difference (−0.09396 to −0.01784) excluded zero (see Figure 3).

**Table 1:** Peak coordinates and sizes of significant whole-brain clusters showing reduced connectivity with the rPCC seed during active stimulation relative to sham.

Brain region*	*x*	*y*	*z*	Cluster size (mm^3^)
Right angular gyrus*	59	−50	44	2,535
Right supramarginal gyrus*	62	−14	40	1,886
Right superior parietal gyrus	32	−38	59	5,992
Right precuneus*	12	−69	23	3,995
Left precuneus*	−2	−50	56	1,984
Left postcentral gyrus	−39	−19	62	3,922
Left posterior superior temporal gyrus*	−64	−28	4	1,753
Left supramarginal gyrus*	−61	−18	31	5,279

Clusters were identified from the voxelwise paired contrast of seed-based functional connectivity maps (sham > rPCC stimulation), using cluster-extent Monte Carlo correction for multiple comparisons (cluster-wise *p* < 0.05; minimum cluster size = 187 mm^3^). The table reports anatomical labels, MNI peak coordinates (*x*, *y*, *z*), and cluster size in mm^3^. Asterisks (*) indicate clusters located within canonical default mode network (DMN) territories. Six of the eight significant clusters fell within the DMN: right angular gyrus, right supramarginal gyrus, left supramarginal gyrus, right precuneus, left precuneus, and left posterior superior temporal gyrusies.

To evaluate whether session order contributed to the observed condition differences, we compared the sham–stimulation effects between participants who received stimulation first and those who received sham first. No order-related differences were observed (all *p*-values >0.58 across clusters), suggesting that the main effect is unlikely to be driven by systematic order confounds.

## Discussion

### Conceptual rationale and causal motivation

Studying the neuroanatomical substrates of self-awareness in humans remains challenging. Correlational evidence links DMN dysfunction with anosognosia in AD, with particular emphasis on the PCC as an early and vulnerable hub. Contemporary models describe the PCC as a highly connected, metabolically active node supporting internally directed cognition and self-referential processing, embedded within partially dissociable DMN subsystems (Raichle et al., 2001; Leech and Sharp, 2014). Despite these associations, direct causal tests of whether perturbing this network can transiently alter connectivity and thereby provide an experimental model of self-related processing remain limited.

The present study sought to establish such a proof of concept by disrupting the DMN via focal stimulation of the right PCC in healthy young adults. A perturbation approach in neurologically intact individuals avoids the interpretive challenges inherent to pathological case series in AD, where diffuse neurodegeneration and compensatory reorganization complicate inference (Eldaief et al., 2011; Beynel et al., 2020). References to self-awareness, metacognition, or anosognosia therefore serve to motivate future extensions of this paradigm and should not be interpreted as constructs directly assessed here.

### Methodological feasibility of deep rPCC perturbation

Targeting the right PCC poses substantial technical challenges due to its deep medial location. Conventional figure-of-eight coils are poorly suited for such targets; accordingly, deep TMS with neuronavigation was employed to concentrate stimulation at the required depth, consistent with bended-coil design studies demonstrating enhanced penetration relative to standard coils (Tadayonnejad et al., 2024; Talebinejad and Musallam, 2010).

Stimulation intensity was calibrated using lower limb motor evoked potentials, as the leg representation of M1 lies at a depth comparable to the PCC. Because several DMN hubs are deeply situated and medial, scalp EEG offers limited sensitivity to network-wide changes; stimulation was therefore combined with resting-state fMRI to capture distributed connectivity effects. Continuous theta burst stimulation (cTBS) was selected given its capacity to induce sustained changes in cortical excitability lasting minutes to hours (Suppa et al., 2016).

Although the target was anatomically specified, deep stimulation is expected to engage a relatively broad medial parietal volume. Coil geometry and neuronavigation were used to constrain field distribution, and the spatially distributed pattern of connectivity reductions supports interpretation in terms of network-level reconfiguration rather than a strictly focal effect.

### Network-level effects of rPCC cTBS

Right-PCC cTBS reduced resting-state functional connectivity between the seed region and multiple DMN clusters, including the right angular gyrus, bilateral supramarginal gyri, bilateral precuneus, and the left posterior superior temporal gyrus. These decreases align with prior evidence that TMS can modulate intrinsic connectivity within and between large-scale systems, including the DMN (Eldaief et al., 2011; Beynel et al., 2020).

Connectivity changes were measured 15.2 min (±3.4 min) after stimulation, within a time window compatible with behavioral assessment. This temporal profile supports the feasibility of pairing perturbation with brief, time-locked tasks in future experiments designed to probe causal links between PCC-centered DMN dynamics and metacognitive processes.

The present findings are restricted to network-level effects. They demonstrate that deep cTBS targeting the right PCC transiently modulates PCC-centered DMN connectivity in healthy young adults, without permitting inference regarding specific cognitive or awareness-related functions.

### Translational implications for self-evaluation and anosognosia

Within this network-level framework, the results provide a rationale for testing whether causal perturbation of PCC-centered DMN connectivity influences self-evaluative processes. In healthy participants, self-awareness can be operationalized using trial-by-trial confidence ratings and performance accuracy during brief tasks administered within the post-stimulation window. Signal detection–based metrics such as meta-d′ and metacognitive efficiency offer performance-independent indices of self-monitoring suitable for this purpose (Maniscalco and Lau, 2012; Fleming and Lau, 2014).

Should right-PCC perturbation selectively alter metacognitive efficiency while preserving first-order accuracy, this would constitute mechanistic evidence linking PCC-centered DMN function to self-evaluation. More broadly, experimentally establishing brain–behavior relationships under controlled perturbation may help bridge systems-level DMN models with clinical phenomena such as anosognosia in AD.

Loss of insight in aging and AD is likely multifactorial, with additional contributions from executive control and salience networks beyond PCC-centered DMN processes (Valera-Bermejo et al., 2022). Clarifying these interactions will require multimodal approaches and refined behavioral paradigms.

### Methodological and statistical considerations, limitations, and future directions

Interpretation should consider several constraints. The absence of a pre-stimulation baseline scan within each session limits the ability to fully dissociate stimulation-induced effects from session-related fluctuations. Although the randomized counterbalanced crossover design and absence of detectable order effects reduce the likelihood of systematic bias, future studies incorporating within-session pre/post acquisitions would provide stronger causal isolation.

Deep TMS necessarily engages a relatively broad cortical volume. Even with neuronavigation, field spread cannot be completely excluded. Individualized electric-field modeling may improve spatial precision in subsequent work.

No behavioral assessment was administered during the post-stimulation window. Consequently, the present study cannot determine whether transient DMN disruption altered self-evaluative processes. Embedding brief, time-locked tasks that quantify both first-order performance and second-order awareness will be essential for establishing direct brain–behavior links.

Unilateral right-PCC targeting was chosen based on prior literature implicating right-hemisphere midline and parietal regions in self-referential processing and reduced awareness in neurological conditions. Lateralization effects remain incompletely resolved and warrant systematic comparison of right, left, and bilateral stimulation protocols.

The sample size was modest; however, the within-subject contrast yielded a robust network-level effect in the expected direction, supporting the feasibility of the perturbation approach. Larger cohorts will refine precision estimates, characterize inter-individual variability, and strengthen generalizability.

ROI-wise effects did not survive familywise correction across all 78 parcels, although they converged directionally with cluster-corrected seed-based findings. Ongoing methodological debate regarding cluster-extent inference highlights the importance of transparent thresholds, nonparametric validation, and replication (Eklund et al., 2016).

## Conclusion

Deep cTBS targeting the right posterior cingulate cortex produced a transient downregulation of connectivity within the default mode network in healthy young adults. Seed-based analyses identified multiple clusters, predominantly within canonical DMN territories, that were less coupled with the rPCC under stimulation than under sham, with convergence across analytic approaches.

These findings establish a feasible human model of transient PCC-centered DMN perturbation and provide a causal framework for future investigations of self-referential and awareness-related processes.

## Data Availability

The datasets presented in this article are not readily available because this study contain sensitive human subject information and cannot be made publicly available due to privacy and ethical restrictions. Data may be made available by the corresponding author upon reasonable request and completion of a data-sharing agreement. Requests to access the datasets should be directed to youssef.bellaali@gmail.com.
